# Rectal cancer management during the COVID-19 pandemic (ReCaP): multicentre prospective observational study

**DOI:** 10.1093/bjs/znab129

**Published:** 2021-05-07

**Authors:** R E Clifford, D Harji, L Poynter, R Jackson, R Adams, N S Fearnhead, D Vimalachandran, the ReCaP Steering Committee and Collaborators, A Vallance, M Evans, H Mohan, N Foley, E O’Connell, J Kinross, A Bhangu, A Acheson, S Moug, J Hughes, K Kong, M Fok, J Wilson, J D Jayasinghe, A Minicozzi, M A Thaha, H Patel, D Smith, M Martinez-Iglesias, S Argyropoulos, J Johnston, A Dosis, F Mosley, R Antakia, I Abu-Nayla, H Joshi, D Westwood, K Dixon, M Al-Azzawi, E Howie, D Watt, A Khan, K Ahmed, W Rea, P Nastro, S Williams, N Solanki, G Akritidis, C Bruce, L Dickerson, S Tewari, G Tewari, V Gupta, N Reay-Jones, J Sagar, S Kudchadkar, N Cirocchi, A Rai, O Al Habsha, S Duff, L Poynter, M Boshnaq, S Saeed, C J Wright, R Dickson-Lowe, M Evans, R Harries, N Spencer, K Parkins, V Bevan, S Bodla, A Ghafar, N Yassin, S Mills, J Law, J Lim, J Sarveswaran, S Dasmohapatra, D Worku, S J Moug, G Kourounis, A Alasadi, S Barman, N Battersby, S Ingram, D Arora, J Lund, P Daliya, M Elzawahry, S Abdul-Hamid, L Weenink, F D McDermott, I R Daniels, N J Smart, E T Walker, S J Muse, R Mirnezami, S Chowdhury, J Gilliland, G Williams, H Jones, H Whewell, D Harji, B Griffiths, A Farquharson, R Mashar, C Weerasinghe, M Ransome, M A Javed, A Samad, S Shamim, E Vitovska, C Hall, C Renfigo, I Maitra, J Norman, C Florance, Y El-Dhuwaib, R Clifford, D Vimalachandran, N Manu, S Kumar, K Altaf, S Ahmed, J Wyatt, M Mesri, J Alberts, N Keeling, A Mishra, N Ward, M Lim, L MacDonald, H S Elshafey, W Awad

**Affiliations:** 1 Institute of Cancer Medicine, University of Liverpool, Liverpool, UK; 2 Population Health Science, Newcastle University, Newcastle upon Tyne, UK; 3 Imperial College London, London, UK; 4 Liverpool Clinical Trials Unit, Liverpool, UK; 5 University of Cardiff, Cardiff, UK; 6 Cambridge University Hospitals NHS Foundation Trust, Cambridge, UK; 7 The Countess of Chester Hospital NHS Foundation Trust, Chester, UK

## Abstract

Concerns over unacceptable high mortality in patients with rectal cancer undergoing surgery or systemic therapy who contract COVID-19 have led to widespread adoption of alternative treatment strategies.The ReCaP study aimed to study these variations and associated outcomes.

## Introduction

Over 8000 patients are diagnosed with rectal cancer in the UK each year^1^. Treatment has improved over recent years as a result of incremental advances in optimized surgical technique, clinical staging, pathological quality control, and multidisciplinary management^2^. Neoadjuvant therapy is often required in patients with locally advanced tumours, and is usually delivered according to a long-course strategy (long-course radiotherapy, LCRT). Hypofractionated short-course strategies (short-course radiotherapy, SCRT) may also be used, traditionally with immediate surgery, but recently have been combined with strategies such as delayed surgery^3^^,^^4^ and/or systemic chemotherapy^5^. Such regimens offer potential benefits for patients in terms of reducing treatment time and access to systemic therapy, but may also be of use in areas with limited healthcare resources or geographical access to specialist services^5^^,^^6^.

The COVID-19 pandemic has created a unique situation in the UK, with multidisciplinary teams balancing the risks of perioperative COVID infection against those of disease progression. Coupled with altered patient behaviour in accessing healthcare, constrained diagnostic and critical care facilities, there has been rapid change in the traditional multimodal treatment strategy for rectal cancer^7^. The aim of the prospective ReCaP study was to follow patients with rectal cancer managed in the UK during the pandemic, and to determine short-term, long-term, and patient-reported outcomes. This article presents the short-term results for the first 500 patients recruited.

## Methods

A multicentre, prospective observational study was performed across the UK from 23 March 2020 (national governmental lockdown) and is ongoing. The study was performed as a substudy of the IMPACT portfolio^8^ through the Association of Coloproctology of Great Britain and Ireland. The primary objective was to determine the short-term clinical and pathological outcomes associated with each management strategy. Anonymized data were collected for short-term outcomes and so ethical approval was not required. Any hospital managing patients with rectal cancer through a formal multidisciplinary team (MDT) was eligible for inclusion; rectosigmoid tumours were excluded.

## Results

The first 500 patients were recruited from 42 sites between 23 March 2020 and 28 September 2020. The demographics of the entire cohort are reported in *Table S1*.

**Fig. 1:**
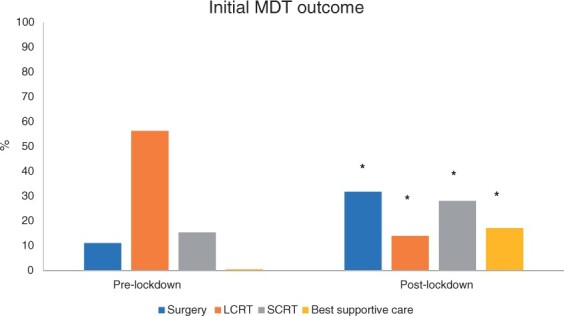
Summary of initial multidisciplinary team outcomes for patients diagnosed before lockdown, during the first national lockdown, and after lockdown LCRT, long-course radiotherapy; SCRT, short-course radiotherapy. *P < 0.005 *versus* before lockdown (T test).

A summary of the initial MDT outcomes for patients, divided into those diagnosed before the date of national lockdown on an established treatment pathway *versus* those diagnosed during and after the lockdown, is shown in *Table 1*. A change in MDT primary outcome owing to COVID-19 was declared for 22.3 per cent of those with a new diagnosis. The temporal change in treatment strategy before, during, and after lockdown is illustrated in *Fig. 1*.

**Table 1:** Summary of initial multidisciplinary team outcomes for patients with a new diagnosis since 23 March 2020 and on an existing treatment pathway

	Diagnosis before lockdown	**Diagnosis during or after lockdown**	Total
	(*n* = 208)	(*n* = 292)	(*n* = 500)
**Primary MDT outcome**			
SCRT	32 (15.4)	82 (28.1)	104 (20.8)
LCRT	117 (56.3)	41 (14.0)	158 (31.6)
Straight to surgery	23 (11.1)	93 (31.8)	116 (23.2)
Organ preservation surgery	3 (1.4)	11 (3.8)	14 (2.8)
Best supportive care	1 (0.5)	50 (17.1)	51 (10.2)
Other	7 (3.4)	13 (4.5)	20 (4.0)
Surgery to metastatic site	1 (0.5)	0 (0)	1 (0.2)
Surveillance	1 (0.5)	6 (2.1)	7 (1.4)
Referral to alternative MDT	5 (2.4)	7 (2.4)	12 (2.4)
**Change in plan owing to COVID-19**	18 (8.7)	65 (22.3)	83 (16.6)

Values in parentheses are percentages. MDT, multidisciplinary team, SCRT, short-course radiotherapy; LCRT, long-course radiotherapy.

Some 114 patients (22.8 per cent) received neoadjuvant chemotherapy alone or as part of a radiotherapy regimen. SCRT and delay increased significantly immediately after commencement of lockdown (from 15.4 to 45.2 per cent; *P* < 0.001), dropping back after its lifting to 19.1 per cent. Fourteen patients received concurrent systemic therapy, with only six (6 per cent) receiving consolidation chemotherapy after SCRT. Eight patients (8.2 per cent) required a stoma before completion of neoadjuvant therapy. At the time of analysis, only 41 patients (39.4 per cent) had undergone surgery after SCRT, with many procedures delayed because of COVID-19 restrictions. A total of 158 patients (31.6 per cent) received LCRT, and the rate significantly decreased after lockdown (from 56.3 to 14.0 per cent; *P* < 0.001). Thirty-three patients (20.9 per cent) required a stoma before completion of LCRT.

To date, 225 patients have undergone surgical resection, 51.6 per cent without any neoadjuvant therapy. Anterior resection (38.7 per cent) or abdominoperineal resection (25.3 per cent) were the most common operations performed. There was a significantly higher rate of admission to level 2 care after operation before compared with after lockdown (36.0 *versus* 10.8 per cent; *P* < 0.001). Some 50.3 per cent of operations were performed or attempted laparoscopically, with a 30-day re-operation rate of 5.8 per cent. Thirty-three patients (14.7 per cent) were deemed to have had a different operative approach. In 49 patients (21.8 per cent) a stoma was created owing to COVID-19 alone, and the overall stomas formation rate was 86.7 per cent. The 30-day mortality rate was 1.3 per cent, with only two patients declared as being COVID-19-positive within 30 days. A best supportive care strategy was adopted in 10.2 per cent of the cohort; the rate was significantly higher in patients diagnosed before lockdown (17.1 *versus* 0.5 per cent).

A comparison of tumour characteristics, and clinical and pathological outcomes of patients who underwent SCRT or LCRT, or proceeded straight to surgery is summarized in *Table 2*.

**Table 2:** Summary of tumour staging, and oncological and pathological outcomes for patients undergoing short-course radiotherapy and delay, long-course radiotherapy, or straight to surgery

	SCRT and delay	LCRT	Straight to surgery
	(*n* = 104)	(*n* = 158)	(*n* = 116)
**Age (years)**			
< 60	19 (18.3)	53 (33.5)	44 (37.9)
60–80	51 (49.0)	97 (61.4)	72 (62.1)
> 80	64 (61.5)	8 (5.1)	0 (0)
**Sex ratio (M : F)**	69 : 35	112 : 46	78 : 38
**ASA fitness grade**			
I–II	76 (73.1)	127 (80.4)	100 (86.2)
III–IV	28 (26.9)	29 (18.4)	16 (13.8)
**Primary tumour category**			
T0	0 (0)	0 (0)	0 (0)
T1	0 (0)	3 (1.9)	9 (7.8)
T2	24 (23.1)	8 (5.1)	49 (42.2)
T3	60 (57.7)	91 (58.0)	49 (42.2)
T4	20 (19.2)	56 (35.4)	9 (7.8)
**Primary node category**			
N0	36 (36.0)	28 (17.8)	0 (0)
N1	40 (40.0)	69 (43.9)	8 (6.9)
N2	24 (24.0)	60 (38.2)	0 (0)
**Primary metastasis category**			
M0	93 (89.4)	138 (87.9)	94 (81.0)
M1	11 (10.6)	19 (12.1)	22 (19.0)
**Threatened CRM**	48 (46.2)	121 (77.0)	22 (19.0)
**Tumour height (cm)**			
Lower (0–6)	56 (53.8)	86 (54.4)	32 (27.6)
Mid (7–11)	35 (33.7)	58 (36.7)	61 (52.6)
Upper (> 12)	13 (12.5)	14 (8.9)	23 (19.8)
**Reported surgical complication rate**	7 (6.7)	40 (25.4)	28 (24.1)
**Radiological outcomes if restaged**			
Complete clinical response	15 (4.4)	10 (6.3)	–
T category regression	36 (34.6)	41 (25.9)	–
N category regression	22 (21.5)	46 (29.1)	–
T category progression	5 (4.8)	6 (3.8)	–
N category progression	4 (3.8)	6 (3.8)	–
M category progression	4 (3.8)	14 (8.9)	–
**Pathological outcomes**			
Pathological complete response	3 (2.9)	16 (10.1)	–
Extramural vascular invasion	7 (6.7)	17 (10.8)	26 (22.4)
Lymphovascular invasion	10 (9.6)	17 (10.8)	27 (23.4)
Perineural invasion	6 (5.8)	13 (8.2)	12 (10.3)
R1 resection	3 (2.9)	9 (5.7)	4 (3.4)
Interval from diagnosis to surgery (days)*	155.5	207	40
Interval from end of radiotherapy to surgery (days)*	86	105	–
**Watch and wait**	23 (22.1)	19 (12.0)	0 (0)
**Best supportive care**	5 (4.8)	2 (1.3)	0 (0)

Values in parentheses are percentages unless indicated otherwise; *values are median. SCRT, short-course radiotherapy; LCRT, long-course radiotherapy; CRM, cirumferential resection margin.

## Discussion

The COVID-19 pandemic presented unprecedented challenges in the treatment of rectal cancer. National lockdown, resource reallocation, and rapidly emerging data showing the unacceptably high morbidity of perioperative COVID-19 resulted in a sudden and dramatic change in multimodal management^9^. Although many of these COVID-adapted strategies have understandably been undertaken out of necessity, their safety remains unclear. However, this dramatic change may offer the potential opportunity for a paradigm shift in the management of rectal cancer^10^.

Following national lockdown, 22.3 per cent of patients underwent a change in initial MDT outcome. There was a rapid shift to hypofractionated radiotherapy regimens, with SCRT increasing from 15.4 per cent to 45.2 per cent during the first 8 weeks, and LCRT dropping from 56.3 per cent to 14.0 per cent. Compared with recently published national bowel cancer audit (NBOCA) data, which reports an overall SCRT rate of just 10 per cent, these rates represent a significant deviation from standard UK practice.

Short-term clinical and pathological outcomes of SCRT appear to be similar to those of LCRT; however, the omission of systemic chemotherapy in many of these patients (80.7 per cent) needs to be monitored carefully^5^. Some 22.1 per cent of patients entered an active monitoring pathway, potentially reflecting the increased rate of T2 tumours in the LCRT cohort (23.1 *versus* 5.0 per cent). Although not all of this cohort underwent surgical resection, the reported pathological complete response (pCR) rate of 2.9 per cent is much lower than the pCR rate of 30 per cent in the recently reported radical surgery versus organ preservation via short-course radiotherapy followed by transanal endoscopic microsurgery for early-stage rectal cancer (TREC) study^11^. Interestingly, the surgical complication rate was much lower (6.7 per cent) than that in the LCRT group (25.4 per cent) or among those who proceeded directly to surgery (24.1 per cent). The reasons for this are unclear, but this result is worthy of note given the potential logistical and geographical benefits SCRT may offer.

Despite concerns over perioperative COVID-19 infection, there was perhaps a surprising increase in the number of patients proceeding straight to surgery. Notably, despite reduced critical care availability, surgery was still associated with low mortality and re-operation rates, comparable to NBOCA data (5.8 *versus* 8.4 per cent for re-operation). This finding is likely to reflect the rapid establishment of green, elective ‘ring-fenced’ beds. Despite concerns over viral transmission and aerosolization during laparoscopic surgery, 50.3 per cent of procedures were still undertaken laparoscopically. Reassuringly, although 19.0 per cent of patients had a threatened circumferential resection margin on preoperative staging, the R1 resection rate was only 3.4 per cent, with 30.7 per cent nodal positivity.

The overall rate of best supportive care was 10.2 per cent across the cohort, but there was significant change between patients diagnosed after *versus* before lockdown (17.1 *versus* 0.5 per cent). The overall rate is lower than the non-operated rate of 39.7 per cent reported in the NBOCA, but includes patients with advanced disease. The reasons behind the dramatic change are unclear, but may represent MDT uncertainty regarding treatment safety and resource allocation^12^, patient choice, or may relate to a more subtle acknowledgement of pessimism in the face of the pandemic regarding patients who would be considered on the borders of a curative *versus* palliative approach.

Limitations of this study include potential site participation, patient selection, and reporting bias. Some 78.6 per cent of the cohort had an ASA fitness grade of I–II, potentially highlighting an under-representation of more vulnerable patients. Cohorts receiving SCRT and delay and LCRT were non-randomized, with poor completion of MRI tumour regression grading by clinicians to enable accurate assessment of radiological response.

This report has demonstrated a rapid and reactive adaptation in the multimodal management of rectal cancer in the UK in response to COVID-19. A move to shorten treatment regimens appears to have been safe in the short term; however, close surveillance should be undertaken by early imaging and clinical review. Although patients may have experienced benefit in terms of organ preservation, and low stoma and complication rates, this has to be balanced against the significant rise in best supportive care and uncertain long-term oncological outcomes. The ReCaP study will continue to monitor this cohort and subsequently managed patients, and provide further long-term oncological and qualitative outcomes.

## Collaborators

Steering Committee: A. Vallance, M. Evans, H. Mohan, N. Foley, E. O’Connell, J. Kinross, A. Bhangu, A. Acheson, S. Moug. Collaborators: J. Hughes, K. Kong (Aintree University Hospital); M. Fok, J. Wilson (Arrowe Park Hospital); J. D. Jayasinghe, A. Minicozzi, M. A. Thaha, H. Patel (Barts Health NHS Trust, London); D. Smith, M. Martinez-Iglesias, S. Argyropoulos (Bolton Foundation Trust, Bolton); J. Johnston, A. Dosis, F. Mosley (Bradford Teaching Hospital Foundation Trust, Bradford); R. Antakia, I. Abu-Nayla, H. Joshi (Cambridge University Hospitals Trust, Cambridge); D. Westwood, K. Dixon (Crosshouse Hospital); M. Al-Azzawi, E. Howie, D. Watt (County Durham and Darlington NHS Foundation Trust); A. Khan, K. Ahmed, W. Rea, P. Nastro (Darent Valley Hospital); S. Williams, N. Solanki (King’s College Hospital, London); G. Akritidis (Princess Royal University Hospital (PRUH), King’s College Hospital); C. Bruce, L. Dickerson (Leighton Hospital); S. Tewari, G. Tewari, V. Gupta, N. Reay-Jones (Lister Hospital, East and North Hertfordshire NHS Trust); J. Sagar, S. Kudchadkar, N. Cirocchi, A. Rai, O. Al Habsha (Luton and Dunstable Hospital); S. Duff (Manchester University NHS Foundation Trust, Manchester); L. Poynter, M. Boshnaq, S. Saeed, C. J. Wright (Maidstone and Tunbridge Wells NHS Trust); R. Dickson-Lowe (Medway Maritime Hospital); M. Evans, R. Harries, N. Spencer, K. Parkins, V. Bevan (Morriston Hospital, Swansea); S. Bodla, A. Ghafar, N. Yassin (New Cross Hospital, Wolverhampton); S. Mills (Northumbria Healthcare NHS Trust); J. Law (Pennine Acute Hospitals NHS Trust); J. Lim, J. Sarveswaran (Pinderfields Hospital); S. Dasmohapatra (Princess Alexandra Hospital NHS Trust); D. Worku (Queen’s Medical Centre, Nottingham University Hospitals NHS Trust, Nottingham); S. J. Moug, G. Kourounis, A. Alasadi, S. Barman (Royal Alexandra Hospital, Paisley); N. Battersby, S. Ingram (Royal Cornwall Hospital); D. Arora, J. Lund, P. Daliya, M. Elzawahry, S. Abdul-Hamid, L. Weenink (Royal Derby Hospital, Derby); F. D. McDermott, I. R. Daniels, N. J. Smart, E. T. Walker, S. J. Muse (Royal Devon and Exeter NHS Foundation Trust, Exeter); R. Mirnezami, S. Chowdhury, J. Gilliland (Royal Free Hospital, London); G. Williams, H. Jones, H. Whewell (Royal Gwent Hospital, Newport); D. Harji, B. Griffiths (Royal Victoria Infirmary/Freeman Hospital, Newcastle upon Tyne); A. Farquharson, R. Mashar (Shrewsbury and Telford Hospital NHS Trust); C. Weerasinghe, M. Ransome (Southport and Ormskirk Hospital); M. A. Javed, A. Samad, S. Shamim, E. Vitovska (St Helens and Knowsley NHS Trust); C. Hall, C. Renfigo, I. Maitra (Stepping Hill Hospital); J. Norman, C. Florance, Y. El-Dhuwaib (Surrey and Sussex NHS Trust); R. Clifford, D. Vimalachandran, N. Manu, S. Kumar (Countess of Chester Hospital, Chester); K. Altaf, S. Ahmed (Royal Liverpool and Broadgreen University Hospital); J. Wyatt, M. Mesri (Warrington and Halton NHS Trust); J. Alberts, N. Keeling, A. Mishra, N. Ward (West Suffolk Hospital); M. Lim, L. MacDonald, H. S. Elshafey, W. Awad (York Teaching Hospitals NHS Trust, York).

*Disclosure.* The authors declare no conflict of interest.

## Supplementary material

Supplementary material is available at *BJS* online

## Supplementary Material

znab129_Supplementary_Data

## References

[1] https://www.nboca.org.uk/resources/nboca-dataset-2019-2020/ (accessed 5 January 2021)

[2] Clifford R , Govindarajah N, Parsons JL, Gollins S, West NP, Vimalachandran D. Systematic review of treatment intensification using novel agents for chemoradiotherapy in rectal cancer. Br J Surg 2018;105:1553–1572 30311641 10.1002/bjs.10993 PMC6282533

[3] Chok AY , Kontovounisios C, Rasheed S, Kelly ME, Aalbers AGJ, Abdul Aziz N et al The impact of the COVID-19 pandemic on the management of locally advanced primary/recurrent rectal cancer. Br J Surg 2020;107:e547–e548 10.1002/bjs.11893 PMC7436568 32779191

[4] Spencer K , Jones CM, Girdler R, Roe C, Sharpe M, Lawton S et al The impact of the COVID-19 pandemic on radiotherapy services in England, UK: a population-based study. Lancet Oncol 2021;22:309–320 33493433 10.1016/S1470-2045(20)30743-9 PMC7825861

[5] Bahadoer RR , Dijkstra EA, van Etten B, Marijnen CAM, Putter H, Kranenbarg EMK et al; RAPIDO collaborative investigators. Short-course radiotherapy followed by chemotherapy before total mesorectal excision (TME) *versus* preoperative chemoradiotherapy, TME, and optional adjuvant chemotherapy in locally advanced rectal cancer (RAPIDO): a randomised, open-label, phase 3 trial. Lancet Oncol 2021;22:29–42 33301740 10.1016/S1470-2045(20)30555-6

[6] Romesser PB , Wu AJ, Cercek A, Smith JJ, Weiser M, Saltz L et al Management of locally advanced rectal cancer during the COVID-19 pandemic: a necessary paradigm change at Memorial Sloan Kettering Cancer Center. Adv Radiat Oncol 2020;5:687–689 32322758 10.1016/j.adro.2020.04.011 PMC7175910

[7] Courtney A , Howell AM, Savva N, Warren O, Kontovounisios C, Tekkis P et al The impact of the COVID-19 pandemic on colorectal cancer service provision. Br J Surg 2020;107:e521–e522 32856751 10.1002/bjs.11990 PMC7461495

[8] Vallance AE , Harji D, Fearnhead NS; IMPACT collaborative. Making an IMPACT: a priority setting consultation exercise to improve outcomes in patients with locally advanced, recurrent and metastatic colorectal cancer. Eur J Surg Oncol 2019;45:1567–1574 31097310 10.1016/j.ejso.2019.04.005

[9] Marijnen CAM , Peters FP, Rödel C, Bujko K, Haustermans K, Fokas E et al International expert consensus statement regarding radiotherapy treatment options for rectal cancer during the COVID 19 pandemic. Radiother Oncol 2020;148:213–215 32342861 10.1016/j.radonc.2020.03.039 PMC7194592

[10] Morris E , Goldacre R, Spata E, Mafham M, Finan PJ, Shelton J et al Impact of the COVID-19 pandemic on the detection and management of colorectal cancer in England: a population-based study. *Lancet Gastroenterol Hepatol* 2021;6:199–208 10.1016/S2468-1253(21)00005-4 PMC7808901 33453763

[11] Bach SP , Gilbert A, Brock K, Korsgen S, Geh I, Hill J et al Radical surgery *versus* organ preservation via short-course radiotherapy followed by transanal endoscopic microsurgery for early-stage rectal cancer (TREC): a randomised, open-label feasibility study. Lancet Gastroenterol Hepatol 2021;6:92–105 33308452 10.1016/S2468-1253(20)30333-2 PMC7802515

[12] Price P , Barney SE. Initiation of the Global Coalition for Radiotherapy during the COVID-19 pandemic. Lancet Oncol 2020;21:752–753 32502440 10.1016/S1470-2045(20)30281-3 PMC7266608

