# Supplementary material for: Rectal cancer management during the COVID-19 pandemic (ReCaP): multicentre prospective observational study
Source: Br J Surg. 2021 May 7;108(11):1270–3. doi: 10.1093/bjs/znab129 (PMC8136009; doi:10.1093/bjs/znab129)
Supplement: znab129_Supplementary_Data [file znab129_supplementary_data.docx]

Supplementary Table 1 – Baseline cohort demographics

| **Characteristic** | **Number of patients (500) (%)** |
| --- | --- |
| Gender  Male  Female | 324 (64.8)  176 (35.2) |
| Age, years  <40  40-49  50-59  60-69  70-79  >80 | 26 (5.2)  34 (6.8)  91 (18.2)  144 (28.8)  132 (26.4)  73 (14.6) |
| Baseline performance status  0  1  2  3  4  5 | 254 (50.8)  161 (32.2)  54 (10.8)  27 (5.4)  3 (0.6)  1 (0.2) |
| ASA  1  2  3  4  5 | 133 ((26.6)  260 (52.0)  93 (18.6)  13 (2.6)  1 (0.2) |
| Normally fit and well | 322 (64.4) |
| Medical comorbidities  Diabetic  IHD, CCF  COPD/Asthma  CKD  Vascular disease  Immunosuppression  IBD | 68 (13.6)  58 (11.6)  51 (10.2)  20 (4.0)  0 (0.0)  15 (3.0)  4 (0.8) |
| BMI >35kgm^2^ | 32 (6.4) |
| Current smoker | 52 (10.4) |
| Previous COVID-19 diagnosis | 8 (1.6) |
| Previous colorectal surgery  Anterior resection  APR  Pelvic exenteration  TEMS  Hartmann’s  Right hemicolectomy  Subtotal colectomy  Other | 41 (8.2)  11 (2.2)  2 (0.4)  2 (0.4)  2 (0.4)  4 (0.8)  3 (0.6)  2 (0.4)  15 (3.0) |

*Table 1. A summary of patient baseline demographics*

*(ASA: American Society of Anaesthesiologists, IHD: Ischaemic heart disease, CCF: Congestive cardiac failure, COPD: Chronic obstructive pulmonary disease, CKD: Chronic kidney disease, IBD: Inflammatory bowel disease, BMI: Body mass index, APR: Abdomino-perineal resection, TEMS: Transanal endoscopic microsurgery)*
